# Acrolein exposure suppresses antigen-induced pulmonary inflammation

**DOI:** 10.1186/1465-9921-14-107

**Published:** 2013-10-16

**Authors:** Page C Spiess, David Kasahara, Aida Habibovic, Milena Hristova, Matthew J Randall, Matthew E Poynter, Albert van der Vliet

**Affiliations:** 1 Department of Pathology, College of Medicine, D205 Given Building, 89 Beaumont Ave, Burlington, VT 05405, USA; 2 Department of Medicine, University of Vermont, Burlington, VT 05405, USA; 3 Department of Environmental Health, Harvard School of Public Health, Boston, MA, 02115, USA

**Keywords:** Cigarette smoke, Electrophile, Inflammation, Asthma, COPD, Nrf2, NF-κB, JNK

## Abstract

**Background:**

Adverse health effects of tobacco smoke arise partly from its influence on innate and adaptive immune responses, leading to impaired innate immunity and host defense. The impact of smoking on allergic asthma remains unclear, with various reports demonstrating that cigarette smoke enhances asthma development but can also suppress allergic airway inflammation. Based on our previous findings that immunosuppressive effects of smoking may be largely attributed to one of its main reactive electrophiles, acrolein, we explored the impact of acrolein exposure in a mouse model of ovalbumin (OVA)-induced allergic asthma.

**Methods:**

C57BL/6 mice were sensitized to ovalbumin (OVA) by intraperitoneal injection with the adjuvant aluminum hydroxide on days 0 and 7, and challenged with aerosolized OVA on days 14–16. In some cases, mice were also exposed to 5 ppm acrolein vapor for 6 hrs/day on days 14–17. Lung tissues or brochoalveolar lavage fluids (BALF) were collected either 6 hrs after a single initial OVA challenge and/or acrolein exposure on day 14 or 48 hrs after the last OVA challenge, on day 18. Inflammatory cells and Th1/Th2 cytokine levels were measured in BALF, and lung tissue samples were collected for analysis of mucus and Th1/Th2 cytokine expression, determination of protein alkylation, cellular thiol status and transcription factor activity.

**Results:**

Exposure to acrolein following OVA challenge of OVA-sensitized mice resulted in markedly attenuated allergic airway inflammation, demonstrated by decreased inflammatory cell infiltrates, mucus hyperplasia and Th2 cytokines. Acrolein exposure rapidly depleted lung tissue glutathione (GSH) levels, and induced activation of the Nrf2 pathway, indicated by accumulation of Nrf2, increased alkylation of Keap1, and induction of Nrf2-target genes such as HO-1. Additionally, analysis of inflammatory signaling pathways showed suppressed activation of NF-κB and marginally reduced activation of JNK in acrolein-exposed lungs, associated with increased carbonylation of RelA and JNK.

**Conclusion:**

Acrolein inhalation suppresses Th2-driven allergic inflammation in sensitized animals, due to direct protein alkylation resulting in activation of Nrf2 and anti-inflammatory gene expression, and inhibition of NF-κB or JNK signaling. Our findings help explain the paradoxical anti-inflammatory effects of cigarette smoke exposure in allergic airways disease.

## Background

Cigarette smoking remains prevalent worldwide, and is among the main preventable causes of pulmonary and cardiovascular disease and death. In addition to strong links with lung cancer, cigarette smoking or exposure to environmental tobacco smoke are also associated with chronic pulmonary inflammatory diseases such as COPD and asthma [1,2]. Strong associations exist between cigarette smoking and the frequency and severity of several respiratory tract infections, such as influenza or tuberculosis, due to its impact on the immune system [3,4]. Additionally, altered immune responses and local oxidative stress within the airways of smokers may be responsible for increased incidence and persistence of respiratory infections and chronic inflammation, which ultimately contribute to the development and/or exacerbations of COPD and allergic airway inflammation [5-7]. Yet, the effects of tobacco smoke on the development and severity of allergic asthma are not as clear. The association between passive smoke exposure and childhood asthma is relatively well established [8-10]. However, other studies have failed to demonstrate an association between smoking and asthma, and smokers were in some cases found to be at lower risk of developing asthma compared to non-smokers or ex-smokers [11,12]. Studies in animal models confirm this dichotomy, and demonstrate that cigarette smoke (CS) can promote allergic sensitization and exacerbate allergic responses [13-15], but can also attenuate allergic inflammation and airway hyperresponsiveness during allergen challenge of previously sensitized animals [16-19].

Although the biological effects of CS are due to many diverse mechanisms, several studies invoke the important contribution of CS-derived reactive oxygen species, primarily based on observations of protective effects of thiol-based antioxidants [2,20,21]. However, the main thiol-reactive agents within tobacco smoke are electrophilic aldehydes and ketones, among which acrolein is believed to be of primary importance [2,22-24]. Mainstream CS contains levels of acrolein over 90 ppm [23], and measurements of acrolein levels in airway secretions or exhaled breath condensate from smokers suggest it can reach 1–10 μM in the lung [25,26]. At doses ranging from 0.2-6 ppm, the effects of acrolein vapor mimic those of CS in inhalation studies [27-29]. Indeed, acrolein exposure exerts suppressive effects on the immune system and inhibits alveolar macrophage responses and function [29-31]. These anti-inflammatory and immunosuppressive effects of acrolein are thought to be due to inhibition of redox-sensitive transcription factors, such as nuclear factor-kappa B (NF-κB) and activator protein-1 (AP-1), through direct alkylation of proteins involved in these pathways [32-36]. Furthermore, our recent studies indicate that acrolein exposure alters alveolar macrophage responses by suppressing classical “M1” macrophage responses and favoring alternative “M2” polarization programs, consistent with similar observations in smokers [37]. Additionally, anti-inflammatory effects of various alkylating agents, such as sulforaphane, curcumin and 15d-PGJ_2_, have been linked to alkylation of kelch-like ECH-associated protein 1 (Keap1), and subsequent activation of the transcription factor Nuclear factor (erythroid-derived 2)-like 2 (Nrf2) (reviewed in [38]), which results in the induction of a number of antioxidant and immunosuppressive genes, including heme oxygenase-1 (HO-1) and glutamate-cysteine ligase (GCL), the rate limiting enzyme in glutathione (GSH) synthesis [39].

Although epidemiological evidence suggests an association between acrolein exposure and increased asthma risk [40], limited reports have addressed the direct role of acrolein in allergic airway inflammation [41]. The studies presented herein were designed to explore the impact of acrolein in a mouse model of allergic asthma. Our findings indicate that acrolein exposure (5 ppm, 6 hrs/day) during allergen challenge markedly attenuates Th2-driven inflammatory responses by inhibiting redox-sensitive inflammatory signaling pathways including NF-κB and by activating Nrf2 and inducing anti-inflammatory genes such as HO-1.

## Methods

### Animals and reagents

Male 6–8 week old C57BL/6 mice were purchased from Charles River (Saint Constant, Quebec). All animal studies were approved by the Institutional Animal Care and Use Committee at the University of Vermont. All chemicals were purchased from Sigma-Aldrich (St. Louis, MO) unless otherwise indicated.

### Ovalbumin sensitization and challenge

**Figure 1:**
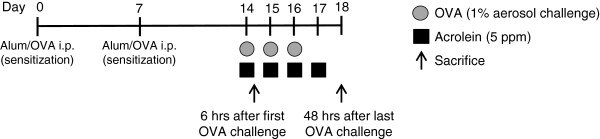
**Schematic diagram of experimental design.** Mice were sensitized to OVA by intraperitoneal injection on day 0 and 7, with 20 μg OVA and the adjuvent aluminum hydroxide (Alum). Sham-sensitized mice received PBS in Alum. Mice were challenged with an aerosolized 1% OVA solution in sterile PBS for 30 min on days 14–16, immediately followed by exposure to acrolein (6 hrs; 5 ppm) on days 14–17. Mice were sacrificed on either day 14 (immediately after acrolein exposure; 6 hrs) or on day 18 (48 hrs after the final OVA challenge).

Mice were injected intraperitoneally (i.p.) with 20 μg of ovalbumin (OVA) solubilized in sterile phosphate buffered saline (PBS) in a 1:1 mixture with the adjuvant aluminum hydroxide (Alum) (Imject® Alum; Thermo Scientific, Rockford, IL) on days 0 and 7 to sensitize to OVA (OVA group) (Figure 1). Sham-sensitized mice received PBS in Alum (Sham group). All mice were exposed to aerosolized 1% OVA solution in sterile PBS for 30 min on days 14–16 and harvested either 6 hrs after the initial challenge on day 14 or on day 18, 48 hrs after the last challenge.

### Acrolein exposures

Mice were placed in a 2 L glass chamber situated in a chemical fume hood and exposed to 5 ppm (11.5 mg/m^3^) of acrolein vapor for 6 hrs/day, on days 14–17 (Figure 1). Vaporized acrolein was diluted with room air to 5 ppm and passed through the chamber at 5 L/min. The acrolein vapor concentration was continuously monitored using an infrared sensor (Miran SapphIRe model M205, Thermo Scientific, Franklin, MA). Control mice were exposed to room air at 5 L/min for 6 hrs/day, on days 14–17.

### Blood collection and OVA-specific IgG1 assay

Mice were sacrificed by i.p. injection of sodium pentobarbital. Following euthanasia, blood was collected via cardiac puncture into serum separator tubes, centrifuged at 3,000 rpm for 10 min, and serum was kept frozen at -80°C until analysis. An ELISA for OVA-specific IgG1 was performed as previously described [42].

### BALF collection and BAL cell counts

After tracheal cannulation, lungs were lavaged 3 times with 500 μL PBS, and lavage fluids were kept on ice until processing. Lung lavage samples were centrifuged at 1500 rpm for 5 min at 4°C. The supernatant was frozen in liquid N_2_ and the cell pellet was resuspended in 400 μL PBS containing 1% bovine serum albumin. Total cell counts were performed using a hemacytometer, and cytospins were prepared for differential cell counts by staining with a modified Wright-Giemsa stain (Protocol Hema 3, Fisher Diagnostics, Middletown, VA). At least 200 cells were counted per slide.

### Airway epithelial cell protein extraction by lysis lavage

Selective removal of airway epithelial cell proteins was performed as previously described [43] following a single OVA challenge and acrolein exposure on day 14 (Figure 1). Briefly, the trachea of each animal was cannulated, the alveolar regions of the lung were blocked by infusion of low-melting-temperature agarose followed by 1% dextrose, and the excised lung was cooled to 4°C, in 5% dextrose for 10 min. The dextrose solution was then removed from the lungs through simultaneous inversion and gentle suction with a syringe, and was repeated until no more solution could be recovered. The airways were then lavaged with 0.5 mL lysis buffer containing 2 M thiourea, 7 M urea, 4% CHAPS, 1% Triton X-100 and 2% Protease Inhibitor Cocktail III (Calbiochem) to recover airway epithelial cell proteins.

### Protein and RNA collection

Following either BALF collection or lysis lavage, lungs were subsequently removed and two right lung lobes were snap frozen in liquid N_2_ for biochemical analysis, while one right lobe was placed in RNAlater (Ambion, Austin, TX) for 24 hrs at 4°C before storage at -80°C for subsequent RNA extraction.

### Lung histology

The left lobe of the lungs was instilled with 4% paraformaldehyde in PBS for 10 min at a pressure of 25 cm H_2_O and placed into 4% paraformaldehyde overnight for fixation of the tissue. Paraformaldehyde-fixed lung lobes were embedded in paraffin and cut into 5 μm thick sections. Sections of paraffin-embedded lungs were deparaffinized and rehydrated to water.

### PAS staining and quantification

Tissue sections were stained for mucus using the Periodic Acid-Schiff (PAS) method, and counterstained with hematoxylin. For quantification of mucus metaplasia, slides were scored using a scale of 0–4 (0 representing no reactivity and 4 being the highest intensity staining observed) for airway Periodic Acid-Schiff reactivity. Each slide was scored by two blinded individuals. The intensity was evaluated for each airway (at least 2) on each section from each animal, and averaged.

### H&E and immunofluorescence imaging

Left lung lobes were processed for hematoxylin and eosin (H&E) staining. Immunofluorescence (IF) staining of lung sections was performed with rabbit polyclonal anti-acrolein (Abcam; 1:500), rabbit anti-serum Club Cell (Clara Cell) Secretory Protein (CCSP; Millipore, Temecula, CA; 1:2000), and secondary antibody goat anti-rabbit Alexa 555 fluorescence conjugated IgG (Invitrogen; 1:500). For nuclear staining, specimens were treated with 4,6-diamidino-2-phenylindole (DAPI). Sections were evaluated by confocal microscopy and analyzed using Metamorph imaging software (v.7.8.2.0; Molecular Devices, Sunnyvale, CA). Representative images of airways were selected for presentation.

### Analysis of cytokine levels

The concentrations of IL-4, IL-13, TNFα, and IL-12p40 were measured in lung lavage supernatants using ELISA, as recommended by the manufacturer (BD Biosciences, San Diego, CA).

### Quantitative reverse transcriptase polymerase chain reaction (RT-PCR)

TRIzol (Invitrogen, Grand Island, NY) and a standard extraction protocol (Qiagen, Germantown, MD) were used to isolate total RNA from lung tissue. Total RNA was then treated with DNase (Qiagen, Valencia, CA) to remove contaminating DNA. Complementary DNA (cDNA) was prepared from 1 μg of total RNA with MMLV reverse transcriptase and Oligo(dT)15 primer (Invitrogen). Quantitative RT-PCR was performed using SYBR Green PCR Supermix (Bio-Rad) and primers designed for various mouse genes (Table 1). *GAPDH* expression was used as a housekeeping gene and relative gene expression was calculated using the 2^-ΔΔCT^ method [44].

**Table 1:** Primers used in the detection of cytokine expression from mouse lung homogenates

**Gene**	**Sense (5′-3′)**	**Antisense (5′-3′)**
*Muc5ac*	AGTCTCTCTCCGCTCCTCTCAAT	CAGCCGAGAGGAGGGTTTGATCT
*Il4*	GGCATTTTGAACGAGGTCAC	ATCGAAAAGCCCGAAAGAGT
*Il13*	AGGAGCTGAGCAACATCACA	GTGGGCTACTTCGATTTTGG
*Il12b*	ACGGCCAGAGAAAAACTGAA	CTACCAAGGCACAGGGTCAT
*Tnf*	TGGAAGACTCCTCCCAGGTA	ACGGCATGGATCTCAAAGAC
*Gclm*	ACCTGGCCTCCTGCTGTGTG	GGTCGGTGAGCTGTGGGTGT

### Transcription factor activity assays

Nuclear extracts were prepared using the Nuclear Extract Kit (Active Motif, Carlsbad, CA) for analysis of DNA binding activity of NF-κB or c-Jun with TransAM NF-κB p65 and TransAM AP-1 c-Jun ELISA kits, respectively (Active Motif, Carlsbad, CA).

### Identification of acrolein-modified proteins by biotin hydrazide labeling

Frozen lung tissues were homogenized in lysis buffer (containing 50 mM HEPES, 250 mM NaCl, 10% glycerol, 1% Triton X-100, 1.5 mM MgCl_2_, 1 mM phenylmethylsulfonyl fluoride, 1 mM EGTA, 2 mM Na_3_VO_4_, and 10 μg/mL of aprotinin and leupeptin) using a tissue homogenizer (Biospec Products, Racine, WI). A total of 300 μg of protein was incubated for 2 hrs with constant mixing with 100 μL of a 50 mM solution of biotin hydrazide (Pierce) (in dimethyl sulfoxide (DMSO), pH 6.0) in a total volume of 200 μL. Samples were placed on ice and incubated for 1 hr with 750 μL of 30 mM NaCNBH_4_ in 1X PBS. Biotin labeled samples were washed 6 times with 300 μL of 20 mM Tris/Cl pH 7.4 and concentrated to 100 μL in 3,000 MWCO filter devices (EMD Millipore, Billerica, MA). To this 100 μL sample, 400 μL of lysis buffer and 100 μL of High Capacity Neutravidin beads were added and mixed constantly overnight at 4°C. The beads were gently pelleted and washed 6 times with 1 mL 0.1 M glycine, pH 2.8. Following a final wash with 1 mL of 20 mM Tris/Cl pH 7.4, samples were boiled for 5 min at 100°C in 100 μL of 2× reducing sample buffer (containing 0.125 M Tris/Cl, 4% SDS, 20% glycerol, 0.47 M β-mercaptoethanol, 0.02% bromophenol blue, pH 6.8), and immediately centrifuged at 14,000 rpm at 4°C for 5 min, whereupon the supernatant containing the biotin-labeled proteins was collected for analysis by Western blotting.

### Analysis of protein thiol content by iodoacetamide-LC-biotin labeling

Lysis lavage samples (100 μg protein) were washed 6x in 3000 MWCO centrifugation devices (Millipore) with 300 μL deoxygenated lysis buffer (50 mM Tris–HCl pH 7.4, 150 mM NaCl, 0.5% (vol/vol) Triton X-100 and 2% protease inhibitor cocktail (Calbiochem)). Samples were concentrated to 100 μL and then labeled with 100 μM (final concentration) iodoacetamide-LC-biotin (Pierce) in DMSO. Samples were mixed for 1 hr at room temp and then mixed 1:1 with 2× reducing sample buffer for Western blot analysis.

### Western blot and cellular GSH analysis

Total lung homogenates, lysis lavage samples, or purified biotin-labeled proteins were analyzed by SDS-PAGE and Western blotting using antibodies against phosphorylated (p) IκBα, p-cJun, cJun, JNK, IKKβ, Nrf2 (D1Z9C) XP®, Keap1 (Cell Signaling, Danvers, MA), IκBα, RelA (Santa Cruz Biotechnology, Santa Cruz, CA), HO-1 (BioVision, Mountain View, CA; 1:1000), and β-actin (Sigma; 1:5000) and detected using HRP-conjugated secondary antibodies (Cell Signaling; 1:1000) or HRP-conjugated streptavidin (Sigma; 1:20,000) and enhanced chemiluminescence (Pierce). Lung homogenates were also used for analysis of reduced GSH [45].

### Statistical analysis

All experiments were performed 2–3 times (with 3–4 animals per treatment group). Data are expressed as mean ± SEM and were analyzed by ANOVA with Tukey correction for multiple comparisons. Results are considered statistically significant if *p* < 0.05.

## Results

### Acrolein exposure suppresses allergen-induced pulmonary leukocyte infiltration and mucus production

**Figure 2:**
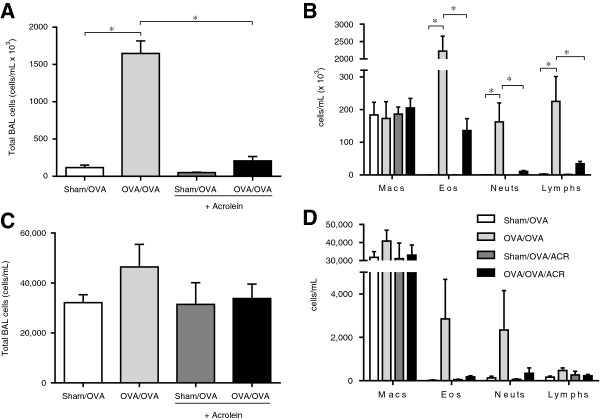
**Acrolein exposure attenuates allergic airway inflammation in OVA-sensitized and challenged mice.** C57BL/6 mice were sensitized and exposed as shown in Figure 1. Bronchoalveolar lavage fluid (BALF) was collected on day 18 **(A,B)** or 14 (6 hrs after OVA challenge; **C,D)** for enumeration of total cells **(A, C)** and differential cell counts **(B,D)**. Results are expressed as mean ± SEM (n = 3-10/group) (*, p < 0.05).

**Figure 3:**
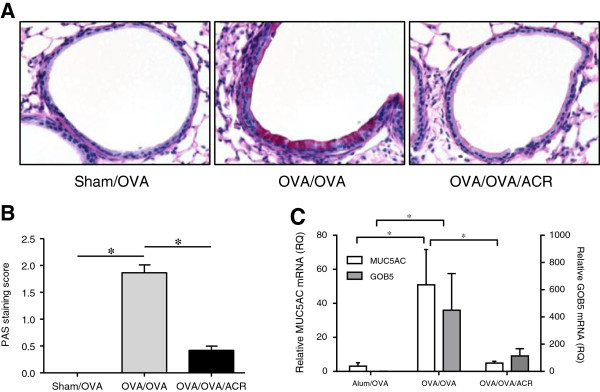
**Acrolein exposure suppresses mucus/goblet cell hyperplasia in response to allergen challenge.** Mucus/goblet cell hyperplasia was evaluated 48 hrs after the last OVA challenge by PAS staining **(A)** which was scored and quantified **(B)**. Lung tissue mRNA gene expression of *Muc5ac* and *Gob5* was analyzed by qRT-PCR **(C)**. Results are expressed as mean ± SEM (n = 4/group) (*, p < 0.05).

To explore the effects of acrolein inhalation on allergic airway inflammation, we used an ovalbumin (OVA) model of asthma and exposed allergen-sensitized mice to acrolein vapor during the OVA challenge phase, and evaluated airway inflammation 48 hrs after the final OVA challenge (Figure 1). As expected, OVA challenge of sensitized mice resulted in allergic inflammation, shown by increased numbers of primarily eosinophils, as well as neutrophils and lymphocytes in BAL fluids. Exposure to acrolein immediately following allergen challenge significantly suppressed these responses, shown by decreased total numbers of BAL cells (Figure 2A), and decreased numbers of eosinophils, neutrophils and lymphocytes (Figure 2B), compared with animals that were not exposed to acrolein. A trend towards suppression of allergen-induced pulmonary cell infiltration was also observed immediately after a single exposure to acrolein following a single OVA challenge (Figure 2C and D), indicating acrolein may have direct and immediate effects on inflammatory pathways. Additionally, acrolein exposure markedly decreased allergen-induced mucus and goblet cell hyperplasia as detected by PAS staining (Figure 3A and B), and significantly decreased mRNA expression of the marker genes *Muc5ac* and *Gob5* (Figure 3C), 48 hrs after the final OVA challenge.

### Acrolein exposure significantly reduces lung cytokine expression and release after challenge

The reduced inflammation observed in antigen-challenged mice that were exposed to acrolein suggests an inhibition of pro-inflammatory cytokines responsible for inflammatory cell recruitment and mucus production. To determine whether acrolein exposure was affecting allergen-induced cytokine production, RT-PCR analysis of cytokine gene expression was performed on lung tissues harvested from animals 6 hrs after a single OVA challenge and/or a single 6-hr acrolein exposure (Table 2). As expected, OVA sensitization and challenge increased lung tissue expression of several Th2 cytokines (*Il4* and *Il13*), and these increases were attenuated following exposure to acrolein. OVA sensitization and challenge also increased expression of *Tnf* (encoding TNFα), but did not significantly affect *Il12b* (encoding IL-12p40). In both cases acrolein exposure significantly suppressed mRNA expression of these genes. Analysis of cytokine release into the airway lumen of animals, 48 hrs after multiple OVA challenge, demonstrated a trend towards increased release of IL-4 and IL-13 in OVA-sensitized mice (Table 3), and acrolein exposure did not significantly affect IL-4 or IL-13 release in these animals. However, OVA sensitization and challenge resulted in a significant increase in IL-12p40 levels in BAL fluid, which was significantly attenuated following acrolein exposure (Table 3). Taken together, these results indicate that acrolein is most likely affecting pathways responsible for the recruitment of inflammatory cells and the mRNA expression and production of pro-inflammatory cytokines in this model of allergic inflammation.

**Table 2:** **Effects of acrolein exposure on inflammatory cytokine mRNA expression from mouse lung homogenates 6 hrs after OVA challenge**^**†**^

**Gene**	**Alum/OVA**	**OVA/OVA**	**Alum/OVA/ACR**	**OVA/OVA/ACR**
*Il4*	1.00 ± 0.20	14.0 ± 4.86^*^	0.94 ± 0.29^#^	5.76 ± 1.42
*Il13*	1.00 ± 0.18	8.91 ± 2.47^*^	1.15 ± 0.37^#^	6.24 ± 1.89
*Il12b*	1.00 ± 0.17	1.28 ± 0.20	0.74 ± 0.09^#^	0.51 ± 0.08^#^
*Tnf*	1.00 ± 0.15	2.46 ± 0.23^*^	0.87 ± 0.13^#^	1.00 ± 0.15^#^

^†^Cytokine levels were measured by RT-PCR of lung homogenates from animals that were sensitized and challenged according to Figure 1. All values are normalized to Alum/OVA controls (mean ± SEM; n = 6–9 mice/group) and ANOVA was used to compare groups (^*^, p < 0.05 vs. Alum/OVA; ^#^, p < 0.05 vs OVA/OVA).

**Table 3:** **Effect of acrolein on Th1/Th2 cytokine levels in BAL fluids 48 hrs after OVA challenge**^**†**^

	**Alum/OVA**	**OVA/OVA**	**OVA/OVA/ACR**
IL-4	33.5 ± 9.3	68.6 ± 27.0	61.6 ± 10.5
IL-13	69.9 ± 28.2	139.7 ± 49.4	100.5 ± 16.1
IL-12p40	60.5 ± 7.7	600.4 ± 66.9^*^	194.0 ± 28.3^#^
TNFα	146.3 ± 41.6	96.1 ± 20.1	191.6 ± 47.6

^†^Cytokine levels were measured by ELISA of lung lavage fluids of animals that were sensitized and challenged according to Figure 1. All values are expressed as pg/mL (mean ± SEM; n = 5–10 mice/group) and ANOVA was used to compare groups (^*^, p < 0.05, vs. Alum/OVA; ^#^, p < 0.05, vs OVA/OVA).

### Acrolein attenuates IgG1 response to repeated OVA challenge

**Figure 4:**
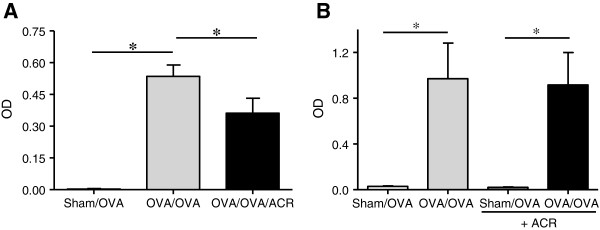
**Acrolein exposure does not directly inhibit OVA-specific serum IgG1 production.** Serum IgG1 was determined by ELISA on day 18 (48 hrs after final OVA challenge; **A)** or day 14 (6 hrs after initial OVA challenge; **B)**. Results are expressed as mean ± SEM (n = 4-6/group) (*, p < 0.05).

To ensure that animals were sensitized to OVA, IgG1 ELISA was performed on serum from animals 48 hrs after the final OVA challenge (Figure 4A) or 6 hrs after the first OVA challenge (Figure 4B). Indeed, all animals sensitized to OVA produced OVA-specific immunoglobulin. An attenuation of the IgG1 response was observed in acrolein exposed animals 48 hrs after the final OVA challenge, but this was not observed in animals that only received a single OVA challenge and acrolein exposure.

### Acrolein exposure results in epithelial cell acrolein-adduct formation and disruption of thiol status

**Figure 5:**
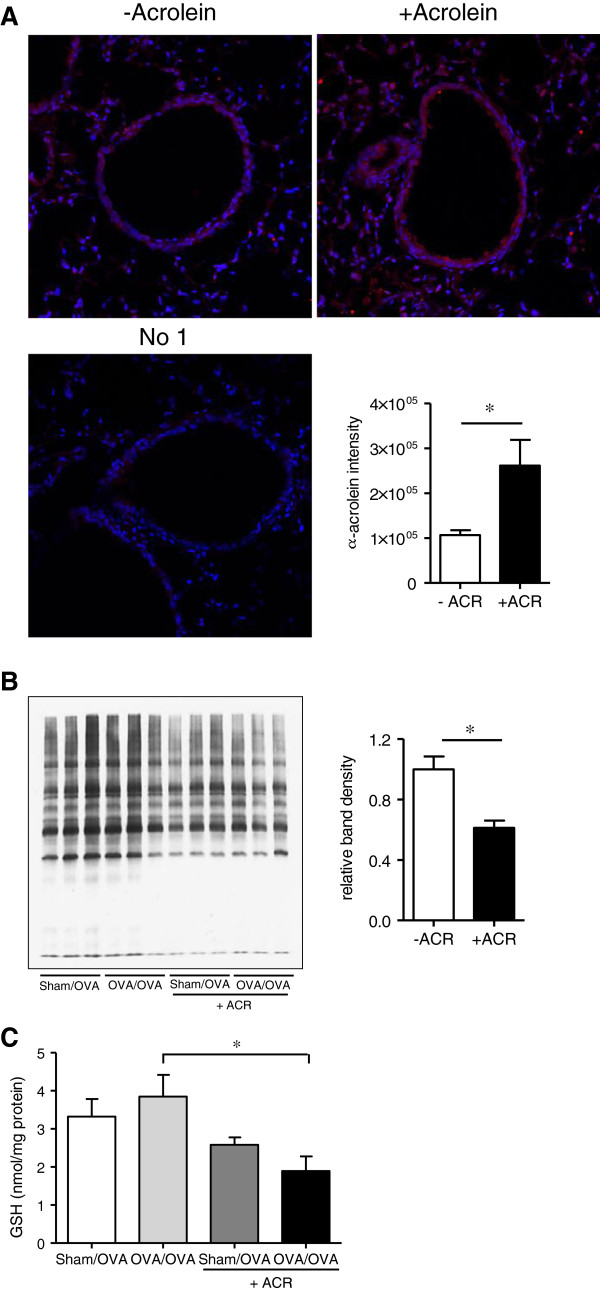
**Acrolein exposure induces protein-adduct formation and disruption of thiol status within the epithelium. (A)** Lung sections were obtained 6 hrs after a single OVA challenge and acrolein exposure (day 14) and probed with anti-acrolein antibody (red) or DAPI (blue), and staining intensity was quantified using Metamorph software A control image without primary antibody (no 1º) is shown to demonstrate specificity. **(B)** Epithelial proteins collected by lysis lavage were labeled with the cysteine-reactive probe Iodoacetyl-LC-biotin, and biotin labeling was visualized by streptavidin blot and quantified using Image J software. **(C)** Reduced GSH was measured by HPLC in lung tissue homogenates. Results are expressed as mean ± SEM (n = 3-6/group) (*, p < 0.05).

Our recent studies demonstrate that acrolein readily and directly interacts with numerous proteins in lung cells [37,46]. To confirm the adduction of inhaled acrolein to airway epithelial cell proteins, IF detection of acrolein-bound protein was performed on lung sections and quantified using Metamorph software. Figure 5A demonstrates that acrolein exposure resulted in significantly increased levels of acrolein-adducted protein in conducting airway epithelial cells compared to controls. Since acrolein primarely reacts with cysteine residues [46], we analyzed the protein cysteine thiol content of lung epithelial cells following acrolein exposure using iodoacetamide-LC-biotin labeling. To this end, airway epithelial cell proteins were obtained by lysis lavage, and labeled with iodoacetamide-LC-biotin to determine thiol content. Successful isolation of airway epithelial cells by lysis lavage was confirmed using both H&E and IF staining of lung sections (Additional file 1: Figure S1). Figure 5B reveals that acrolein exposure leads to a significant loss of epithelial cell protein thiol reactivity compared to non-acrolein exposed controls. The main mechanism of acrolein detoxification involves its conjugation to GSH, which can lead to disruption of cellular redox homeostasis, especially after acute exposure to high concentrations. Indeed, while OVA exposure of sensitized mice did not alter lung GSH status, acrolein exposure resulted in reduced lung tissue GSH levels when measured directly after acrolein exposure, especially in mice that were also challenged with OVA (Figure 5C). No such changes in lung tissue GSH were found 48 hrs after OVA and/or acrolein exposures (results not shown), indicating that GSH depletion by acrolein is transient, and is restored by induction of GSH synthesis. Taken together, these results demonstrate that acrolein exposure leads to formation of acrolein-adducts and an acute loss of both protein and non-protein thiols within airway epithelial cells.

### Acrolein exposure activates the Nrf2 pathway

**Figure 6:**
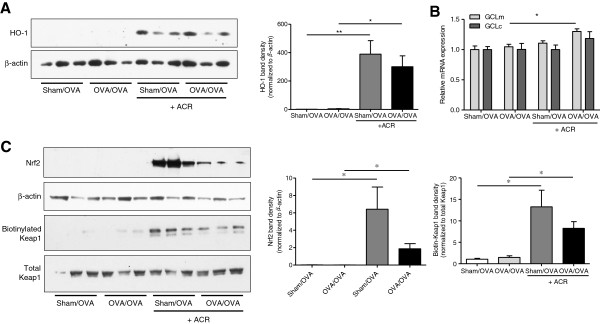
**Acrolein exposure activates the Nrf2 pathway.** Lung tissue homogenates or lysis lavage samples were collected 6 hrs after a single OVA challenge and/or acrolein exposure. **(A)** Analysis of HO-1 protein levels in lysis lavage samples by Western blot*.***(B)** PCR analysis of *Gclm* and *Gclc* in lung tissues. **(C)** Evaluation of biotin hydrazide-labeled proteins and whole lung lysates for the Nrf2 repressor Keap1. Band densities were quantified using Image J software. Results are expressed as mean ± SEM (n = 3-6/group) (*, p < 0.05).

The transcription factor Nrf2 plays a key role in redox homeostasis by regulating the activation of glutathione synthesis and antioxidant defense genes, such as HO-1 [47]. To determine if Nrf2 was activated within the airway epithelium following acrolein exposure, lysis lavage samples from animals exposed to a single OVA challenge and acrolein exposure were analyzed for Nrf2-target protein HO-1. Indeed, acrolein exposure resulted in significant induction of HO-1 in airway epithelial cells (Figure 6A). Nrf2 also transcriptionally regulates the catalytic (*Gclc*) and regulatory (*Gclm*) subunits of glutamate-cysteine ligase, the rate limiting enzyme in GSH synthesis [48] and acrolein exposure was found to increase lung tissue mRNA expression of *Gclm* and *Gclc*, reaching statistical significance in acrolein-exposed OVA/OVA mice vs non-exposed OVA/OVA mice in case of *Gclm* (Figure 6B). We next determined Nrf2 protein accumulation in whole lung tissue lysates, as an indicator of Nrf2 activation, and observed significant increases in Nrf2 in lungs from acrolein-exposed mice, in both sham- and OVA-sensitized animals (Figure 6C). Consistent with the observed accumulation and apparent activation of Nrf2, we observed increased carbonylation of Keap1 in lung homogenates following acrolein exposure as detected by biotin hydrazide labeling and Western blot analysis (Figure 6C), indicating direct alkylation of Keap1 by acrolein through Michael addition to its cysteine residues [49]. Interestingly, the extent of acrolein-induced Nrf2 accumulation and Keap1 alkylation appeared to be reduced in OVA-sensitized and challenged mice compared to sham-sensitized mice, although this did not reach statistical significance. Together, these results indicate that acrolein exposure results in alkylation of Keap1 and thereby leads to activation of the Nrf2 pathway in airway epithelial cells, resulting in increased expression of antioxidant and anti-inflammatory genes, which could contribute to the inhibitory effects of acrolein on OVA-induced allergic inflammation.

### Acrolein exposure inhibits allergen-induced activation of the NF-κB pathway

**Figure 7:**
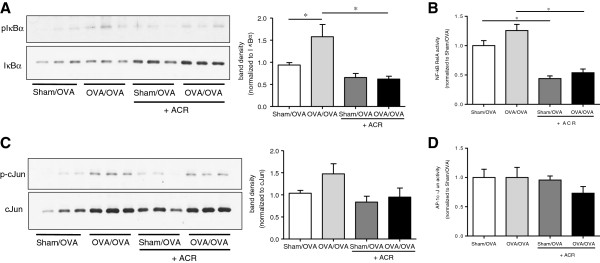
**Inhibition of NF-κB and JNK signaling pathways by acrolein exposure.** Lung tissue homogenates, collected 6 hrs after a single OVA challenge and/or acrolein exposure, were evaluated for phosphorylation of IκBα **(A)**, DNA binding activity of the RelA subunit of NF-κB **(B)**, phosphorylation of c-Jun **(C)** or c-Jun DNA-binding activity **(D)**. Blots were quantified using Image J software, and results are expressed as mean ± SEM (n = 3-9/group) (*, p < 0.05).

OVA-induced inflammation requires the activation of the NF-κB and c-Jun N-terminal kinase (JNK)/AP-1 pathways, and both pathways were previously shown to be affected by components of cigarette smoke [34,37,50]. Analysis of phosphorylation of IκBα, the inhibitor of NF-κB, indicated significantly increased NF-κB activation following OVA sensitization and challenge, and this was significantly attenuated following acrolein exposure (Figure 7A). Using DNA-binding activity assays on nuclear extracts from lung tissues, we evaluated the activation of NF-κB 6 hrs after a single OVA challenge with or without acrolein exposure. As shown, although OVA challenge did not significantly enhance overall NF-κB activation, acrolein exposure inhibited NF-κB binding activity in both sensitized and sham mice (Figure 7B), demonstrating the ability of acrolein to inhibit NF-κB signaling. Activation of JNK signaling was evaluated by analysis of phosphorylation of c-Jun. Although OVA challenge did not significantly increase c-Jun phosphorylation above control levels, acrolein exposure tended to inhibit c-Jun phosphorylation in OVA-challenged mice (Figure 7C). Similarly, while OVA challenge did not significantly increase overall c-Jun nuclear DNA-binding activity, acrolein exposure tended to reduce its activity (Figure 7D). Collectively, these findings indicate that OVA challenge is associated with activation of NF-κB and perhaps JNK, and that acrolein exposure inhibits the NF-κB pathway, and also appears to inhibit the JNK pathway.

### Acrolein adduction of proteins within the NF-κB and JNK pathways

**Figure 8:**
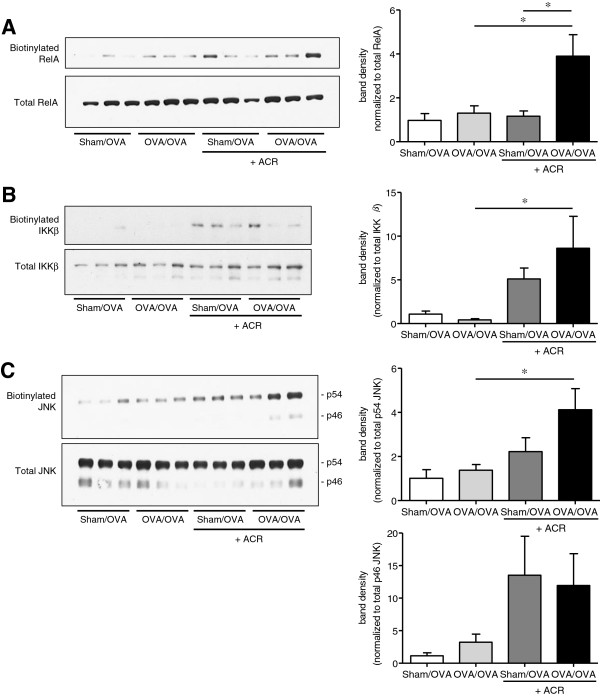
**Acrolein adduction of proteins involved in NF-κB and JNK signaling.** Lung tissue homogenates, collected 6 hrs after a single OVA challenge and/or acrolein exposure, were analyzed for carbonylated proteins by biotin hydrazide labeling and avidin purification. Purified biotinylated proteins were evaluated by Western blot for RelA **(A)**, IKKβ **(B)** or JNK **(C)**. Representative blots are shown. Band intensities were quantified using Image J and expressed as mean ± SEM (n = 6/group) (*, p < 0.05).

Previous findings indicate that acrolein can inhibit NF-κB and JNK signaling by direct alkylation of critical proteins associated with these pathways [33-35,37,51]. To address this possibility, we evaluated acrolein-induced protein carbonylation by Michael addition in lung homogenates of animals subjected to a single OVA challenge and/or acrolein exposure, using biotin hydrazide labeling and avidin purification of biotinylated proteins [52]. Indeed, acrolein exposure significantly increased detectable biotinylation within the NF-κB protein RelA in lungs of OVA-sensitized and challenged mice (Figure 8A), which may explain the decreased DNA binding activity observed in Figure 7B. Since acrolein exposure was found to inhibit OVA-induced phosphorylation of IκBα (Figure 7A), we also determined whether acrolein exposure led to modification of IκB kinase beta (IKKβ), the enzyme responsible for IκBα phosphorylation. Acrolein exposure indeed led to significantly increased levels of biotin hydrazide-labeled IKKβ in lungs of OVA-sensitized and challenged mice, indicating direct alkylation of this protein (Figure 8B). Similarly, as shown in Figure 8C, increased amounts of biotin-labeled JNK isoforms were detected in lung homogenates of animals that were exposed to both OVA and acrolein, which we recently associated with inhibition of JNK signaling [37]. Collectively, these findings suggest that the inhibitory effects of acrolein on the NF-κB and JNK pathways are at least partly due to direct modification by Michael addition of critical proteins within these pathways.

## Discussion

It is well documented that tobacco smoke exposure can contribute to the development of allergic asthma [5,53], and can worsen asthma symptoms and enhance corticosteroid resistance [54,55]. However, a number of studies have also shown that smoke exposure suppresses some mediators of allergic inflammation such as eosinophilia [16,17]. The biochemical mechanisms by which cigarette smoking contributes to lung disease are highly complex, but various lines of evidence support an important role of reactive aldehydes, such as acrolein, found within cigarette smoke [40,56]. Indeed, acrolein exposure in rodent models can induce airways hyperresponsiveness [57] or mucus metaplasia [58,59], important hallmarks of asthma. In addition, based on previous studies demonstrating that acrolein exposure can suppress innate Th1-driven immune responses [36,37,51], we speculated that such actions may promote Th2-polarized inflammatory responses during allergic inflammation. However, our present results indicate that acrolein exposure of allergen-sensitized mice immediately after allergen challenge significantly inhibits overall allergic airway inflammation, cytokine responses, and mucus metaplasia. Our findings are also consistent with a number of studies showing that exposure of OVA-sensitized mice to CS both during and after OVA challenge suppresses eosinophilic inflammation and Th2 cytokine responses [16,60], and imply that these anti-inflammatory effects of CS may in part be due to acrolein.

Acrolein exposure was found to suppress both Th1 cytokines (e.g. IL-12p40) and Th2 responses in the present studies. Therefore, the observed suppression of allergic inflammation and mucus metaplasia were not due to Th2 suppression by activation of Th1 responses, but rather to its more general anti-inflammatory properties. Since the cytokine analysis was performed on BAL fluids, measured cytokines most likely originated primarily from alveolar macrophages, extravasated immune cells, and dendritic cells. Indeed, since dendritic cells may be a major source of IL-12p40, its reduction may reflect inhibited dendritic cell responses. Similarly, the suppressive effects on allergic inflammation may also be related to the ability of acrolein to inhibit T cell responses and proliferation [31].

Because of its strong chemical reactivity, our studies were focused on acute mechanisms by which acrolein affects critical processes involved in regulating airway inflammation. First, acrolein can stimulate anti-inflammatory responses by activating the Nrf2 pathway through direct interaction with redox-sensing cysteine residues within its inhibitor Keap1 [49]. Accordingly, our findings of increased alkylation of Keap1, accumulation of Nrf2 protein, and induction of the Nrf2-regulated genes HO-1 and GCL, indeed indicate the involvement of Nrf2 activation in response to acrolein exposure, and suggest that acrolein activates this pathway by direct modification of Keap1. However, acrolein may also activate Nrf2 by activating kinase signaling pathways to promote phosphorylation of Nrf2, critical for its migration into the nucleus and transcriptional activity [39]. The importance of Nrf2 in allergic inflammation has been addressed in studies with Nrf2-deficient mice, which show enhanced inflammation and airway hyperresponsiveness in a similar OVA model of allergic asthma [61]. Additionally, Nrf2-deficient mice were also found to be more vulnerable to the oxidative and inflammatory effects of chronic cigarette smoke exposure [62]. Various reports indicate that severity of asthma or COPD is associated with impaired Nrf2 activation and function, due to chronic oxidative stress or post-translational modification of Nrf2 [63-65], and chemical activators of Nrf2, such as food-derived electrophilic compounds (e.g. sulforaphane, curcumin), are thought to have therapeutic benefit [39]. Our studies would suggest that acrolein might similarly suppress allergic inflammation, since it has anti-inflammatory properties similar to other electrophiles [37]. However, its chemical reactivity differs from many other anti-inflammatory electrophiles which may be responsible for its toxic properties or ability to promote inflammation or airways hyperresponsiveness [41,66]. Also, while acute exposure to cigarette smoke or acrolein might suppress inflammation due to Nrf2 activation, this may not apply to more chronic conditions in which the defensive capabilities of the Nrf2 pathway may be impaired.

Various lines of evidence indicate the importance of epithelial NF-κB in allergic airway inflammation, chemokine/cytokine-production and mucus metaplasia in allergic inflammation [67,68], and a number of previous findings indicate that acrolein can suppress NF-κB signaling due to modification of redox-sensitive cysteine residues within this pathway [33,34,36]. Indeed, our present results indicate that acrolein exposure inhibits OVA-induced IκBα phosphorylation and nuclear translocation of RelA, and we obtained direct evidence that acrolein exposure led to increased carbonylation of both RelA and IKKβ, suggesting their direct alkylation by acrolein, which is in accordance with previous studies [34,35,37,51]. Thus, acrolein exposure appears to suppress NF-κB signaling both at the level of IKKβ, a critical redox-sensitive kinase within the NF-κB activation pathway [69], and at the level of RelA to inhibit its DNA binding activity [34].

Allergic inflammation may also involve activation of JNK [70] and AP-1 transcription factor family members, which regulate the expression of genes involved in a number of cellular functions including inflammation and pulmonary defense [71]. We previously demonstrated that acrolein can form adducts with specific cysteines within JNK2, which may play a prominent role in the immunosuppressive effects of acrolein [37]. Our present studies indicate that acrolein exposure increased alkylation of JNK isoforms, which was associated with apparent suppression of OVA-induced c-Jun phosphorylation and c-Jun DNA binding activity.

Taken together, our findings indicate that acrolein can suppress allergic airway inflammation by activating Nrf2 and inhibiting major inflammatory signaling pathways related to direct alkylation of critical redox-sensitive proteins in these pathways. It is important to emphasize that acrolein exposure does not suppress inflammation by a single specific mechanism, but by a combination of protein modifications that collectively result in anti-inflammatory responses, due to activation of Nrf2 as well as inhibition of NF-κB and JNK signaling. This property is not unique to acrolein but is shared by many biologically relevant anti-inflammatory electrophiles, including suggested Nrf2 activators such as sulforaphane, that can also inhibit NF-κB and JNK signaling through direct modification of redox-sensitive cysteines [37]. In addition to these direct anti-inflammatory actions, acrolein exposure may also impact on allergic inflammation by more indirect systemic actions, such as the release of stress hormones such a corticosterone [72]. Although these anti-inflammatory actions of acrolein might be interpreted as protective in the context of inflammatory diseases such as asthma, it is prudent to point out that acrolein is reactive with a broader spectrum of biological targets compared to other anti-inflammatory electrophiles [66], and these alternative actions may be responsible for its significant toxic properties and adverse health effects and ability to induce airways hyperresponsiveness.

## Conclusions

In summary, our studies indicate that acrolein may account for the reported anti-inflammatory effects of cigarette smoke in allergic asthma, and highlight multiple and diverse mechanisms by which acrolein exerts such anti-inflammatory actions, through inhibition of NF-κB and JNK pathways and activation of Nrf2 and subsequent anti-inflammatory gene induction. We believe that our findings are mostly relevant in the context of active smoking, which has sometimes been associated with improved asthma symptoms, and may in fact help explain the beneficial actions of the past use of “asthma cigarettes” in treating asthma symptoms (e.g. [73]). Moreover, our findings are also important in helping understand the alterations in inflammatory/immune processes within smokers with asthma, which may for a large part be due to the actions of acrolein. Future studies using acrolein-specific antibodies as a diagnostic tool will be instrumental in addressing the importance of acrolein in CS-related respiratory diseases, and may help assess the importance of acrolein-metabolizing enzymes, such as GSH *S*-transferase P1 [74], in asthma development or severity.

## Abbreviations

CS: Cigarette smoke; NF-κB: Nuclear factor-kappa B; AP-1: Activator protein-1; Keap1: Kelch-like ECH-associated protein 1; Nrf2: Nuclear factor (erythroid-derived 2)-like 2; HO-1: Heme oxygenase-1; GCL: Glutamate-cysteine ligase; GSH: Glutathione; i.p.: Intraperitoneally; OVA: Ovalbumin; PBS: Phosphate buffered saline; Alum: Aluminum hydroxide; ELISA: Enzyme-linked immune sorbent assay; PAS: Periodic acid Schiff; H&E: Hematoxylin and eosin; IF: Immunofluorescence; CCSP: Club Cell (Clara Cell) Secretory Protein; DAPI: 4,6-diamidino-2-phenylindole; IL: Interleukin; DMSO: Dimethyl sulfoxide; JNK: c-Jun N-terminal kinase; IKKβ: IκB kinase beta.

## Competing interests

The authors declare that they have no competing interests.

## Authors’ contributions

PCS participated in the design of the study, experimentation, performed statistical analysis and drafted the manuscript. DK participated in the design of the study, experimentation, performed statistical analysis, and critically revised the manuscript. AH carried out some of the mouse experiments, sample analyses and performed the immunostaining and microscopy. MH participated in the sample collection and analysis. MJR participated in the experimentation and sample collection. MEP participated in the interpretation of the data and critically revised the manuscript. AvdV contributed to conception of the study, participated in interpretation of the data and critically revised the manuscript. All authors read and approved the final manuscript.

## Supplementary Material

Additional file 1: Figure S1 Isolation of airway epithelial cells by lysis lavage. Epithelial cells were selectively removed using the lysis lavage technique. Untreated lungs **(A and C)** and lungs having undergone lysis lavage **(B and D)** were stained with H and E **(A and B)** or immunofluorescence **(C and D)** for Club Cell (Clara Cell) Secretory Protein (CCSP) (red) and DAPI (blue). Click here for file
