# Supplementary figures and images for: Acrolein exposure suppresses antigen-induced pulmonary inflammation
Source: Respir Res. 2013 Oct 16;14(1):107. doi: 10.1186/1465-9921-14-107 (PMC3852782; doi:10.1186/1465-9921-14-107)

## Slide 1
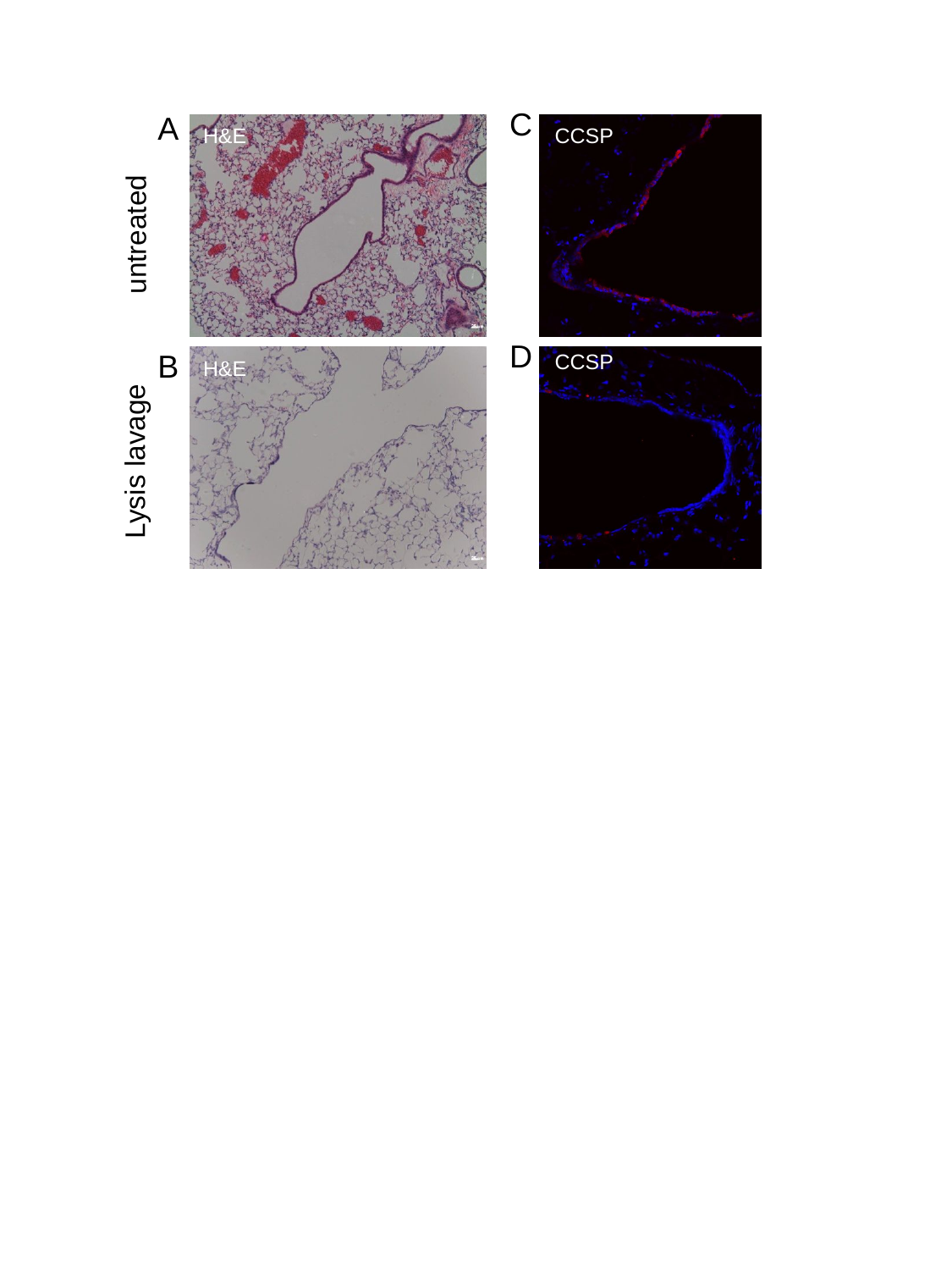

C
A
H&E
CCSP
untreated
D
B
CCSP
H&E
Lysis lavage

Supplement: Additional file 1: Figure S1 — Isolation of airway epithelial cells by lysis lavage. Epithelial cells were selectively removed using the lysis lavage technique. Untreated lungs (A and C) and lungs having undergone lysis lavage (B and D) were stained with H and E (A and B) or immunofluorescence (C and D) for Club Cell (Clara Cell) Secretory Protein (CCSP) (red) and DAPI (blue). [file 1465-9921-14-107-S1.pptx]
